# Smart hydrogels for in situ tissue drug delivery

**DOI:** 10.1186/s12929-025-01166-2

**Published:** 2025-07-24

**Authors:** Shih-Ho Lin, Shan-hui Hsu

**Affiliations:** Institute of Polymer Science and Engineering, National Taiwan University, No. 1, Sec. 4 Roosevelt Road, Taipei, 106319 Taiwan, ROC

**Keywords:** Hydrogels, Drug delivery, Controlled release, Smart hydrogels

## Abstract

The application of smart hydrogels has become a booming research frontier in biomedical engineering. With the development of intelligent drug delivery systems, various biomimetic and biodegradable hydrogels are employed for localized drug delivery to tissues in the preclinical applications. These advanced materials are designed to match the diverse environmental and functional requirements of various tissue types and organs. This article discusses the attractive characteristics of smart hydrogels as delivery systems and reviews the design of a range of smart hydrogels, as well as the challenges of tissue-specific drug delivery, focusing on the last 5 years of frontward research.

## Highlights

- Principles of general hydrogel design
- Synthesis and development of smart hydrogels
- Hydrogel drug delivery to specific tissues
- Current status and challenges of drug delivery from hydrogels to tissues

## Introduction

Hydrogels are water-swollen three-dimensional (3D) networks formed of polymers, small molecules, or colloidal particles, either chemically or physically cross-linked. Within their water-expanded and internal connecting structure, hydrogels are appealing substances for precise therapeutic agent release due to their ability to encapsulate biologically active compounds, such as clinical drugs, proteins, or genes [1]. Their increasing interest as drug delivery systems (DDS) is attributed to their biomimetic properties [2]. Nowadays, hydrogels represent an attractive category of biomaterials applied in various subclasses of medicine and biomedical fields, including cartilage and bone regeneration, biosensors, electronics, and soft robots [3]. The growing emphasis on personalized drug therapy and precision medicine has fueled the innovation of functional hydrogels.

Stimuli-responsive hydrogels are known as a subtype of smart biomaterials, where external triggering factors such as reactive oxygen species (ROS), pH, temperature, electric, sonic, and magnetism, photo, and biomolecules [4]. Moreover, there is a growing fascination with hydrogels endowed with the unique capability to self-repair upon sustaining damage, adding a new dimension to the field. This emerging class of hydrogels, distinguished by its biomimetic healing properties, represents a significant subset within the realm of smart hydrogels. The physical properties of these smart hydrogels undergo abrupt changes in response to minute external stimuli or internal dynamic responses [5]. These adaptabilities extend beyond mere mechanical change, offering a glimpse into the potential for tailored and responsive materials that can dynamically adjust to specific environmental cues.

Smart hydrogels can respond to a variety of triggering factors to achieve complex release profiles. The uniqueness of these hydrogels lies in their nonlinear feedback [3]. The use of smart hydrogels in drug delivery systems can reduce dosing frequency, maintain the therapeutic concentration required in a single dose, adjust release behavior order, and minimize drug side effects by preventing drug accumulation in non-target tissues [6]. Furthermore, the preparation of smart hydrogels is straightforward, making them an ideal choice for drug-incorporated sustained-release systems [7]. Combined with the easy drug loading, high biocompatibility and degradability of the hydrogel itself, this type of smart hydrogel with integrated characteristics has been extensively studied in specialized organs in recent years, such as brain, eyes, bone and cartilage, heart, etc. [8, 9]. Since the first appearance of the “smart hydrogel” keyword in 1991, more than 3,100 related papers have been published [3]. This review provides a detailed overview of various synthetic strategies, release mechanisms, and specific tissue engineering applications of intelligent hydrogels.

## Synthesis strategy of hydrogel

**Fig. 1:**
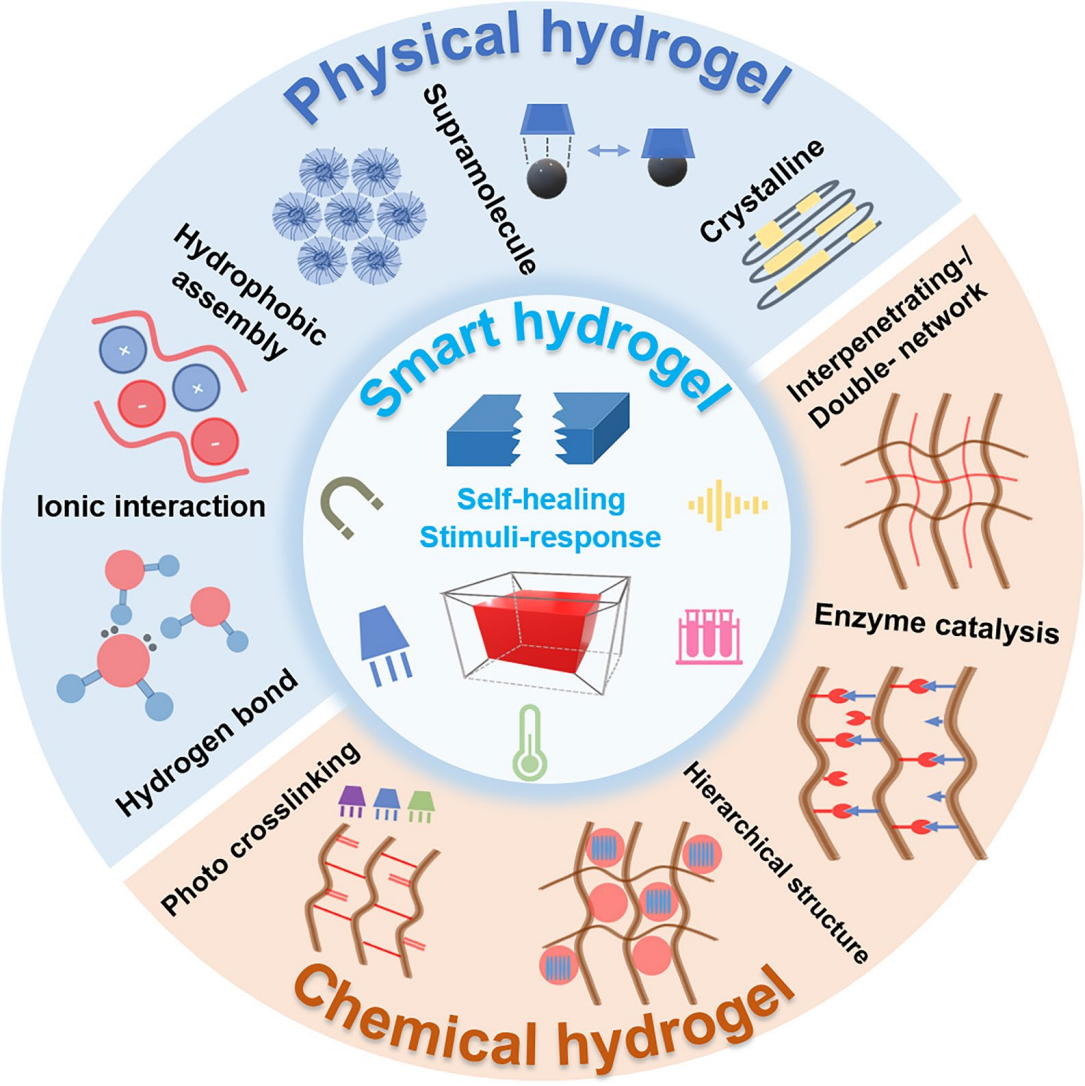
Chemistry and mechanisms for rational design of drug delivery hydrogel and biomedical applications

Hydrogel is composed of a network-like backbone structure with a great number of water molecules (usually more than 50% by weight) bounded together through the intrinsic hydrophilicity of the network structure. Various criteria have been proposed to classify different hydrogels, including the source (naturally derived or synthetic), mechanism (self-assembly or crosslinking), structural conformation, and so on [10]. In this work, hydrogel synthesis strategies are categorized into physically and chemically crosslinked polymer networks. The schematic diagram of the rational design of these hydrogels is shown in Fig. 1. For each type, the corresponding crosslinking mechanisms, stimuli-responsiveness, application potential, and possible improvement strategies are systematically summarized in Table 1.

**Table 1:** Classification and comparative analysis of smart hydrogels based on crosslinking mechanisms, stimuli-responsiveness, and functional modification strategies

Categories	Mechanisms	Stimuli	Advantages	Disadvantages	Strategies
Physical hydrogel	Hydrogen bonding	pH, temperature	Cheap, widespread	Poor stability/performance, swelling	Double networks, interpenetrating network
	Ionic interaction	pH, electron	Biointeraction, rapid formation	Mechanical performance	Crosslinking site, double networks
	Hydrophobic assembly	Temperature, ionic	Microstructure, strong reversibility	Aggregation, ionic precipitation	Hydrophilic-hydrophobic ratio, molecule enhancer
	Host–guest interaction	Chemical factors	Controllability, fast response	Selectivity, expensive	Natural source molecular than synthesis
Chemical hydrogel	Photon crosslinking	Photo, thermo, pH	Precision	Light penetration, initiator toxicity	Optical optimization, size design
	Enzyme catalysis	Chemical factor, ROS	Mild conditions, high specificity	Selectivity, high cost, long reaction time	Microbial enzyme source, microencapsulation
	Click chemistry	Chemical factor, pH, photo	Fast, specificity	Reaction conditions, functional group	Water-soluble system development
	Hierarchical network	Multi-responsive	Strong mechanical properties	Complex synthesis, scale up difficulty	Precise structural design

### Physical hydrogel

Physically crosslinked hydrogels (physical hydrogel) are typically constructed from colloidal factors, micelles, particles, and proteins, as well as secondary forces, including hydrogen bonds, electrostatic forces, supramolecular forces, stereo-complexing, and hydrophobic self-assembly [11]. In general, they are formed through spontaneous self-assembly and stacking processes. The repeated units rely on compact stacking to form a continuous network structure, raising higher solid contents versus chemically crosslinked hydrogels. The individual interactions within physical hydrogels are inherently weak, yet their abundance gives rise to complex behaviors, such as temperature-induced phase transitions or shear-induced rearrangements [12].

#### Hydrogen bonding

Hydrogen bonding is an attractive interaction between an electron donor (usually hydrogen) and an electron acceptor (e.g., the lone pair on nitrogen, oxygen, and fluorine). Polyvinyl alcohol (PVA) hydrogel is a typical hydrogel purely formed through hydrogen bonding. The PVA hydrogels are produced in vitro by freeze–thaw cycles [13]. Many types of polymer can be used to generate hydrogen bond-based hydrogels, such as poly(N,N-dimethylacrylamide), poly(acrylic acid), and poly(N-isopropylacrylamide) [14, 15]. While a single hydrogen bond is weak compared to other physical associations and covalent bonds, it plays an important role when it becomes numerous. This is the common case for biological systems. Deoxyribonucleic acid (DNA) polymeric chains can spontaneously assemble into well-defined secondary or higher-ordered structures based on hydrogen bonding. Since Matsuda and Nagahara first utilized DNA as linkers in water-soluble copolymers for the preparation of hydrogels in 1996, a large number of DNA hydrogels have been proposed [16]. Meanwhile, hydrogen bonds are also widely used in conjunction with all the other linkages, such as chemical acylhydrazone linkages [17].

#### Ionic/electrostatic interactions

Polyelectrolyte complex hydrogels are formed through the self-assembly process involving the complexation of oppositely charged block polymers [18]. The synthetic approach for generating electrostatic hydrogels stems from block copolymers with blocks of differing electrical charges, blending polymers with anti-electricity, or adding charged small molecules to establish inter/intra-electrostatic networks. A prototypical instance of electrostatic hydrogels in biological applications is the self-assembly of alginate biopolymers and divalent calcium ions [19]. These naturally derived charged polysaccharides (other examples include chitosan, hyaluronic acid, xanthan, pectin, and so on) exhibit biodegradability and biocompatibility. In the pharmaceutical domain, polysaccharides have served as excipients in tablet formulations and been utilized in their hydrogel forms to develop modulated drug delivery systems [20]. Besides, electrostatic hydrogels frequently play a role in pH-responsive and electric field-responsive designs, as well as in the development of self-healing hydrogels [21].

#### Hydrophobic assembly

Hydrophobic assembly constitutes another pivotal mechanism in the formation of hydrogels. Hydrophobic interactions occur among non-polar regions of molecules, leading to the self-assembly of hydrophobic segments. In the pharmaceutical domain, a well-known synthesized block copolymer is poly(ethylene glycol)-b-poly(propylene oxide)-b-poly(ethylene glycol), commonly marketed as Pluronic^®^ or Poloxamer^®^. The gelation of Pluronic hydrogel occurs when the concentration and temperature surpasses the critical threshold, resulting from the tight accumulation of micelles [22]. The modulation of hydrophobicity by temperature control of the hydrophobic segment causes the hydrogel micelles to aggregate, with transitions occurring at body temperature. Self-assembled hydrogels prepared through hydrophobic assembly offer an expandable and tunable platform for drug delivery applications [22]. In another case, Yuan et al. proposed a poly(D, L-lactide-co-glycolide)–poly(ethylene glycol)–poly(D, L-lactide-co-glycolide) hydrogel to deliver vancomycin for treating osteomyelitis [23]. Owing to the presence of hydrophobic cavities, these self-assembled hydrogels find widespread applications in the delivery of small-molecule drugs and proteins. However, their drawback lies in large alteration of their properties in response to changes in pH, ionic, and humid environments.

#### Host–guest supramolecular interaction

Supramolecular hydrogels formed through recognition-directed host–guest interactions showcase enhanced attributes such as superior self-healing property, injectability, and stimuli-responsiveness. These desirable characteristics stem from the dynamic and reversible nature of the involved interactions, typically correlating to the hydrophobic effect. The host–guest interaction involves a macrocyclic molecule with a cavity and a corresponding guest molecule, capable of forming complexes through distinctive size complementarity and non-covalent interactions [24]. In addition, the host–guest complexation features a higher level of selectivity and better binding strength than other physical interactions, leading to well-defined stoichiometry, spatial network, and self-healing rate. Cyclic oligosaccharides represent an important family for acting host molecules, including cyclodextrins with their three most abundant variants (α-, β-, and γ-CD, containing 6, 7, and 8 d-glucose units, respectively). Mixing host–guest supramolecular hydrogels can form shear-thinning, self-healing guest hydrogels for applications as injectable tissue scaffolds, drug delivery vehicles, and bioprinting inks [24]. Recently, Xu et al. engineered an injectable host–guest drug delivery system for the controlled release of two different drugs simultaneously [25].

### Chemical hydrogel

Chemically crosslinked hydrogels (chemical hydrogels) are constructed by covalent bonds between polymer chains, providing greater stability, better resistance to hydrolysis, and longer degradation than the physical ones. They exhibit excellent mechanical properties and enhanced stability under physiological conditions. The construction of polymeric covalent networks can be achieved through a one-step reaction of their own functional groups. This category is commonly referred to as "click" chemistry, which represents a subtype of reactions characterized by high efficiency, excellent specificity, biological orthogonality, and mild reaction conditions. Another category involves catalytic reactions through ultraviolet light exposure or enzyme mediation. Furthermore, these hydrogels can simultaneously incorporate multiple reactions to construct double networks, semi-interpenetrating networks, and hierarchical structures. These structured hydrogels often participate in the preparation of smart and responsive hydrogels.

#### Photon crosslinking

Photo-crosslinking using UV- or visible light is one of the most thorough explorations for creating covalently crosslinked hydrogels. By simply adjusting parameters such as light intensity, exposure time, and illumination area, photopolymerization enables excellent control over the spatiotemporal formation and network characteristics of hydrogels. Various groups, including methacrylic acid, maleic acid, and thiol-ene, have been employed to modify a wide range of main chain molecules, such as biopolymers like gelatin, hyaluronic acid, and cellulose, or numerous synthetic polymers, to prepare photo-crosslinked hydrogels [26]. Gelatin methacryloyl (GelMA), a commonly used photopolymerized hydrogel system, is extensively utilized in regenerative applications for bone, heart, cornea, epidermal tissue, cartilage, blood vessels, etc. [27]. Moreover, Ruoyu et al. proposed a GelMA hydrogel system incorporating drug-loaded liposomes, which exhibited triple controlled phase-release capabilities. The features included early release of deferoxamine, mid-term release of bovine serum albumin and bone morphogenetic protein-2, and long-term release of paclitaxel. The system demonstrated high efficacy in the formation of new and mature lamellar bone in a rat femoral condyle defect model [28].

#### Enzyme catalysis crosslinking

Enzyme-catalyzed reactions represent a key biological process in the construction of supramolecular hydrogel networks for biomedical applications. The chemical selectivity, regional specificity, and stereoselectivity of enzymes play crucial roles in adjusting the structure of hydrogels and substrates through enzymatic catalysis. Enzyme-mediated crosslinked hydrogels mimic the extracellular matrix, exhibiting unique physicochemical properties and functions such as water retention, biodegradability, biocompatibility, biological stability, and bioactivity. Commonly employed enzymes in these reactions include tyrosinase, horseradish peroxidase, transglutaminase, lysozyme, etc. [28]. The crosslinking mechanism of tyrosinase is based on the oxidation of phenolic groups (tyrosine, catechol, and polyphenols) in its active site. As one example, a tyrosinase-crosslinked silk fibroin and gelatin hybrid hydrogel incorporating calcium ions was prepared to investigate the calcium release behavior of the fabricated hydrogel in bone tissue regeneration, which also emphasized the ability of the designed hydrogel to enhance osteogenic differentiation of human bone marrow-derived stromal matrix cells [29].

#### Click chemistry

Click chemical crosslinking is a synthetic category with high yield, no by-products, fast reaction rate, simple reactions, and good biocompatibility. Typically, click chemistry includes nitride-alkyne cycloaddition, thiol-ene, Diels–Alder reaction, and Schiff-based. Zhang and his team exploited the nucleic acid click chemistry to develop a DNA hydrogel combining the DNA drug doxorubicin (DOX) and demonstrated that the drug works better in hydrogels [30]. Furthermore, Ding et al. developed a pH-responsive chitosan hydrogel via thiol-ene UV crosslinking. The swelling and shrinkage of the in vivo formed hydrogel can actively modulate the opposite release behaviors of DOX and bovine serum albumin in mediums of different pH [31].

#### Hierarchical and heterogeneous network hydrogel

The utilization of hierarchical and heterogeneous network hydrogels provides a novel and diverse range of delivery modalities in drug delivery. This approach effectively integrates specific drug properties, such as hydrophobic, high molecular weight, and strongly charged characters, into hydrogels and showcases distinctive release behaviors. Common strategies employed in this context include chemical variation, hybridization of nanoscale composites (micelles, nanoparticles, or liposomes), and microscale architectures [32]. In a reported work, Hsu et al. engineered an adaptable microporous hydrogel consisting of microsized building blocks with opposite charges. This design creates the gradient growth factor concentration to facilitate the propagation of neuraxial growth. The approach has proven efficacious in promoting cell migration and inducing significant bridging effects on peripheral nerve defects [33]. More recently, Lin et al. developed a hierarchical chitosan hydrogel incorporating numerous micellar structures to encapsulate dual drugs with opposite affinity to water. The resulting dual drug-loaded hydrogel exhibits an asynchronous delivery feature, meeting the requirement to address distinct pathological stages in the context of intracerebral hemorrhage stroke [34].

### Smart hydrogel

Smart hydrogels are a major category of advanced hydrogels that have emerged in the past decade. They have great biomedical applications due to their bionic functionalities such as self-healing, shape memory, and environmental responsiveness. Smart hydrogels with various chemically and structurally responsive properties/structures perform excellently in different environmental conditions (e.g., pH, temperature, light, electric fields, magnetic fields, chemical stimulation, and biological factors). Smart hydrogels exhibit different crosslinking, swelling, degradation, and reorganization behavior under stimulation. Through controllable characteristics, smart hydrogels have demonstrated greater intelligence in drug delivery. In the meantime, it is common to integrate multiple smartness into a single hydrogel system as an approach for synergetic therapy.

#### Photo-responsive hydrogel

**Fig. 2:**
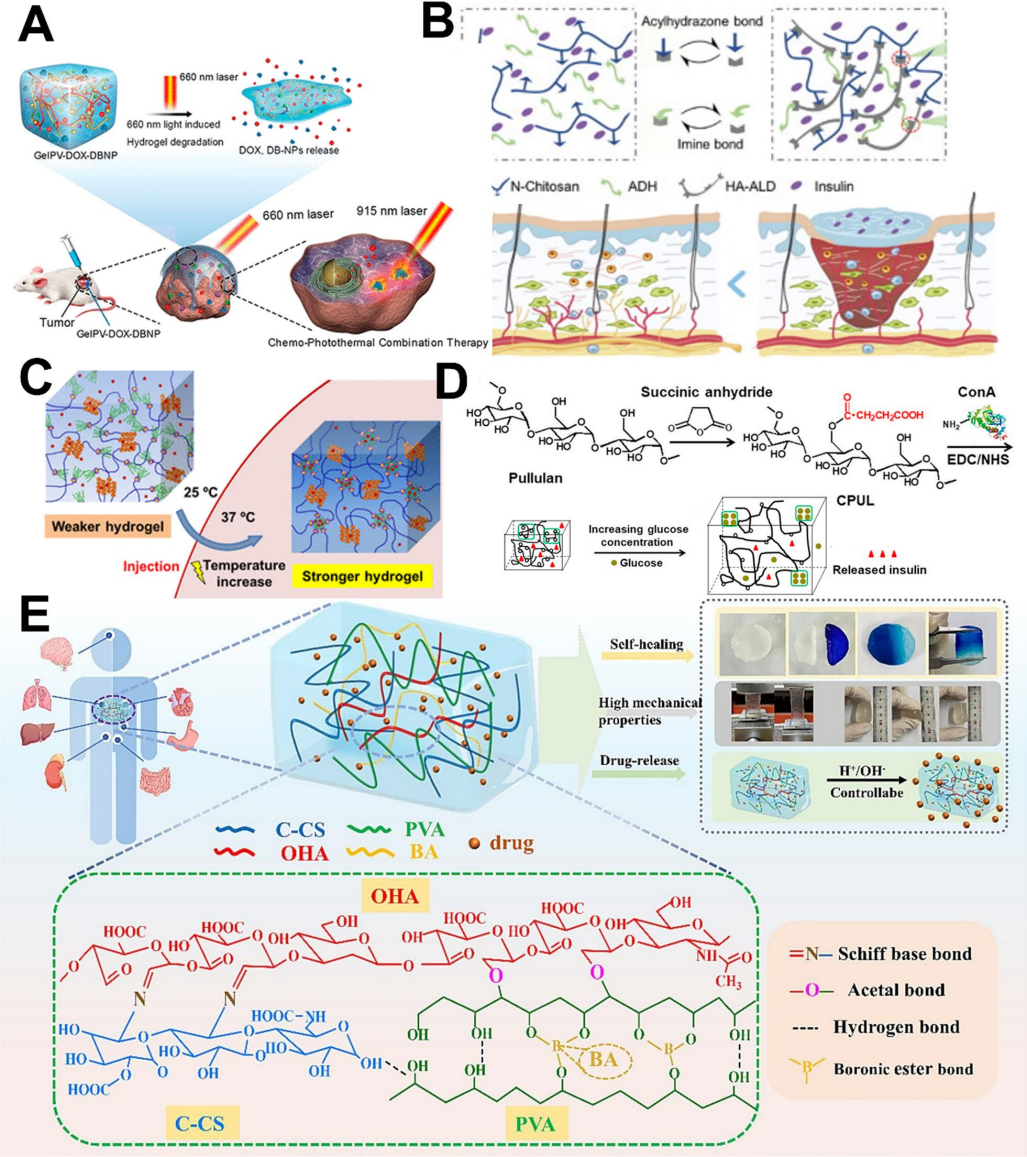
Illustration of smart hydrogels designed for controlled drug release. **A** Photo-responsive and dynamic-covalent hydrogel featuring NIR-triggered drug delivery for photothermal combination therapy. [36] (Copyright 2020 American Chemical Society) **B** pH-responsive hydrogel loaded with insulin as a bioactive dressing to improve diabetic wound healing. [37] (Copyright 2021 Elsevier) **C** Thermo-responsive hydrogel induced by dual supramolecular assemblies and its controlled release property for enhanced delivery of anticancer drugs. [38] (Copyright 2020 American Chemical Society) **D** Glucose-sensitive hydrogels derived from covalently modified carboxylated pullulan and concanavalin A for intelligent controlled release of insulin. [39] (Copyright 2019 Elsevier) **E** A dual dynamic covalent bond hydrogel based on carboxymethyl chitosan with enhanced mechanical properties, high drug encapsulation efficiency, and sustained release properties. [40] (Copyright 2023 Elsevier)

Light irradiation can trigger photo-responsive hydrogels, leading to changes in structure and conformation. Drug release from photo-responsive hydrogel can be achieved through three main mechanisms, including photoisomerization, photochemical reaction, and photothermal reaction [35]. Common light sources include ultraviolet (UV), visible (Vis), and near-infrared (NIR) lights. Although UV crosslinking remains a prevalent approach, the cytotoxic effects of UV on cells and tissues have prompted researchers to favor the utilization of Vis and NIR light sources [36]. As shown in Fig. 2A, a new dynamic covalent fructose-based hydrogel with Vis- and NIR-triggered drug release behavior was reportedly used for photodynamic therapy combined therapy. DOX and photothermal nanoparticles were released under 660 nm light irradiation. For photothermal therapy, the photothermal nanoparticles were subsequently triggered by exposure to 915 nm light which converted 915 nm light irradiation into heat.

#### pH-responsive hydrogel

pH-responsive hydrogels, characterized by acidic or basic functional groups capable of ionization in response to pH fluctuations, exhibit swelling or shrinking behavior, facilitating site-specific drug release. As illustrated in Fig. 2B, a dynamically bonded pH-responsive hydrogel comprising N-carboxyethyl chitosan, hyaluronic acid aldehyde, and adipic acid dihydrazide was reported. Encapsulation of insulin glargine within these hydrogels resulted in sustained and pH-responsive release for up to 14 d, preserving the original biological activity of the insulin. Efficacy assessment was successfully conducted using full-thickness foot wounds in diabetic rats [37].

#### Thermo-responsive hydrogel

Thermo-responsive hydrogels utilize temperature as an external trigger to undergo sol–gel transition, employing polymers that have a phase transition temperature (either upper or lower critical solution temperature) close to body temperature to achieve controlled drug release. Upon temperature elevation, these polymers undergo structural alterations to adjust the viscoelasticity and phase stage. As shown in Fig. 2C, a dual supramolecular host–guest hydrogel composed of poly(N-isopropylacrylamide) star polymer with a β-cyclodextrin core and adamantyl-terminated poly(ethylene glycol) polymer was documented. The copolymers dissolved within the hydrogels can undergo micellization and continue to encapsulate hydrophobic drug carriers, facilitating sustained and gradual release of the anticancer drug (DOX) at the body temperature [38].

#### Chemically responsive hydrogel

Chemically responsive hydrogels are characterized by the integration of functional groups and crosslinked structures. These hydrogels commonly incorporate acidic and alkaline, ionic, and reducing groups, which undergo chemical reactions upon exposure to external chemical stimuli. Additionally, they contain redox-active groups capable of reversible oxidation or reduction in response to changes in redox conditions. This phenomenon leads to alterations in the physical or chemical properties of the hydrogel, facilitating the rapid release of encapsulated drugs at the targeted site. Moreover, enzyme- and glucose-responsiveness is extensively employed in hydrogels owing to the versatility, compatibility, and safety within biological systems. As depicted in Fig. 2D, Lin et al. presented a glucose-sensitive concanavalin hydrogel synthesized through an amidization reaction, enabling intelligent smart controlled release of insulin in reaction to fluctuations in glucose concentration for diabetes therapy [39].

#### Self-healing hydrogel

Self-healing hydrogels, as another major category of smart hydrogels, can self-organize and restore their original mechanical properties after damage. They use either dynamic covalent bonds (Schiff base, Diels–Alder, disulfide bridge, etc.) or a large number of secondary forces (hydrogen bonds, hydrophilic and hydrophobic forces, electrostatic forces) to perform a repeatable damage repair process. Self-healing hydrogels have bionic repair properties and can extend the service life of the materials, so they have received a lot of attention and have been studied and used to develop a variety of smart drug release carriers. Recently, a dual dynamic covalent bond hydrocolloid with Schiff base and borate ester was developed to enhance the mechanical properties, and simultaneously to have high drug encapsulation efficiency and sustained release properties [40], as shown in Fig. 2E.

## Smart hydrogel for in situ delivery and therapy

**Fig. 3:**
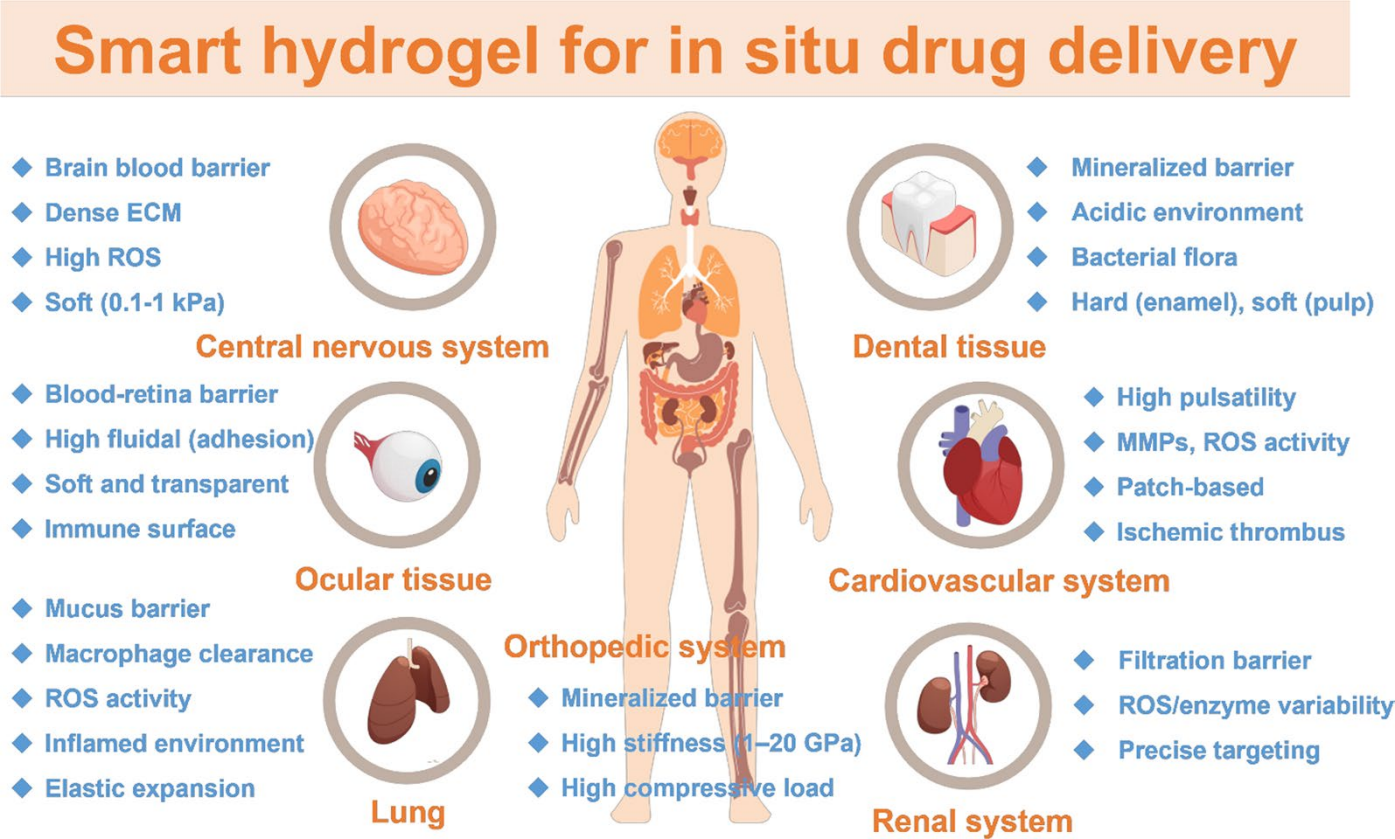
The schematic diagram of drug-loaded smart hydrogels applied to various tissues, organs, and systems. The blue markers indicate the hydrogel properties that are required for application to that tissue

**Fig. 4:**
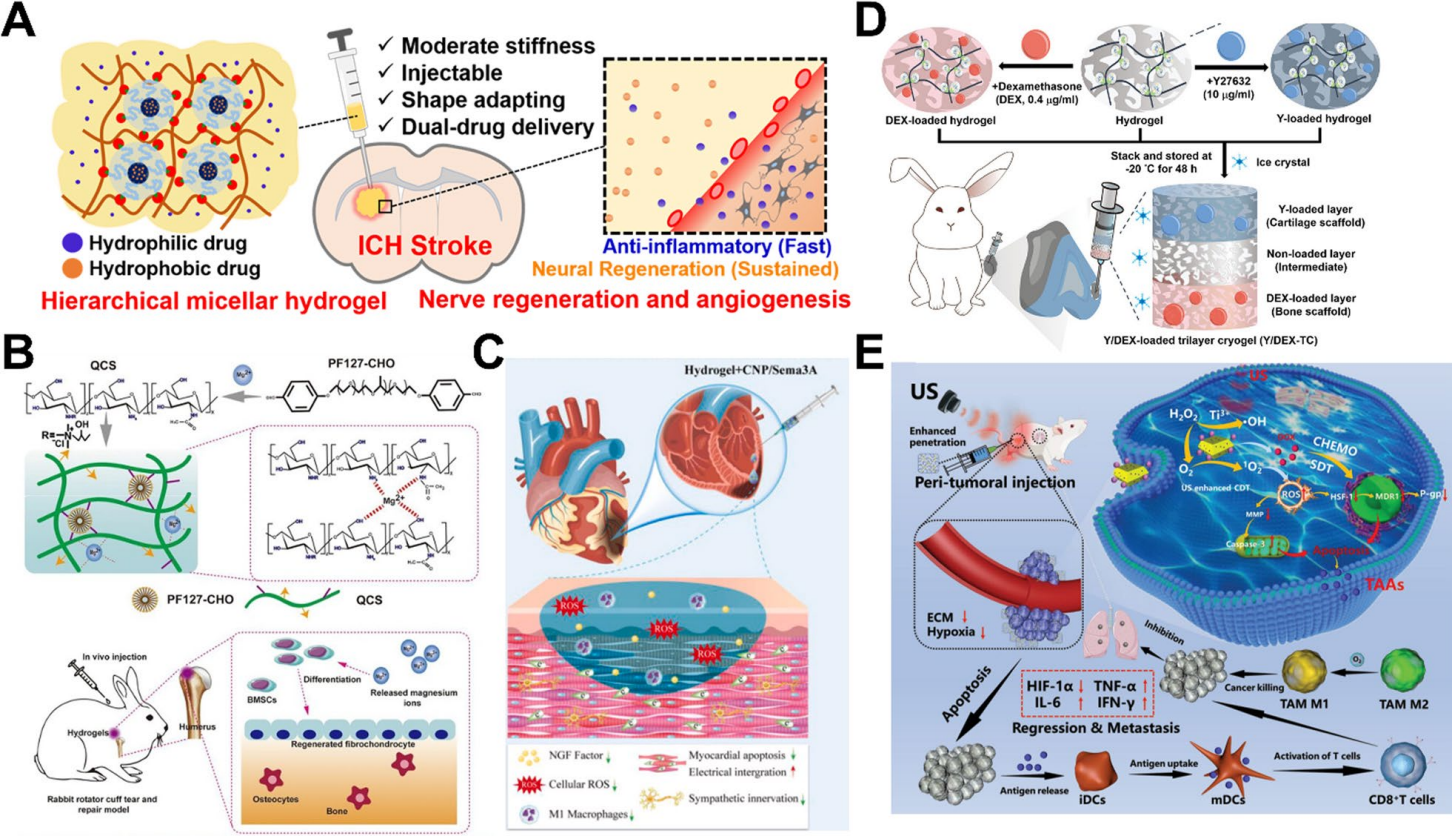
Studies of smart hydrogel systems for in situ drug delivery applications. **A** Self-healing and hierarchical hydrogel for asynchronous delivery targeting intracerebral hemorrhage stroke rats. [34] (Copyright 2023 Wiley) **B** Self-healing adhesive hydrogel shows sustained release of magnesium ions and promotes chondrogenic differentiation for fibrocartilaginous interface regeneration in the rabbit rotator cuff tear model. [48] (Copyright 2020 Elsevier) **C** pH- and ROS-responsive hydrogel with sympatho-immune regulation and cardiac remodeling properties for treating rat myocardial infarction. [51] (Copyright 2025 Elsevier) **D** Self-healed trilayer cryogel with tunable pore sizes and Y27632/dexamethasone releasing ability for rabbit osteochondral regeneration. [49] (Copyright 2024 Wiley) **E** Sono-responsive hydrogel enabling high tissue penetration and anti-tumor immunotherapy. [53] (Copyright 2024 Wiley)

As many new smart hydrogels have been developed and manufactured into advanced drug delivery systems (DDS) in recent years, these highly functional drug-loaded hydrogels are considered to have great preclinical therapeutic prospects. Applying drugs directly to target tissues through smart hydrogels can help improve local treatment effects and reduce systemic side effects. Compared with oral or intravenous injections, systemic administration often results in low treatment efficiency and high toxicity due to blood distribution dilution, metabolic elimination, and non-specific distribution. Smart hydrogels can form stable drug reservoirs in the lesion area through stimulation such as temperature, pH, or enzymes, achieving long-term, controlled release, significantly improving efficacy and reducing the impact on other organs. They are particularly suitable for diseases that require long-term or high-concentration local treatment (such as tumors, chronic inflammation, etc.). This chapter lists smart hydrogel studies aimed at animal experimental testing models before preclinical, including drug release needs and challenges for hydrogel therapy to various tissues. This chapter discusses the in situ tissue delivery of hydrogel with a higher practical value, including central nervous system, ophthalmology, orthopedics and dentistry, and cardiovascular system. Figures 3, 4, and Table 2 summarize the studies on smart hydrogels for in situ tissue delivery applications during the last 5 years (2020–2025).

**Table 2:** Literatures on the application of smart hydrogels for in situ drug delivery during the recent 5 years (2020–2025)

Targets	Hydrogel	Smartness	Model	Cargos	Ref./Year
Central nervous system	Chitosan/Pluronic F127	Self-healing/thermo-responsiveness	Stroke rats	Edaravone/Minocycline hydrochloride	[34], 2023
	PNIPAM/nanocrystals	Thermo-responsiveness	Parkinson’s rats	Magnolol	[54], 2021
	PLGA/PEG	Thermo-responsiveness	Alzheimer's rats	Curcumin	[55], 2022
	Chitosan/Pluronic F127/ black phosphorus	Thermo-responsiveness	Alzheimer's mice	Methylene blue	[42], 2024
Ocular	PEG-DA/PNIPAM	Thermo-responsiveness	Healthy rhesus macaques	Aflibercept	[44], 2020
	Carboxymethyl chitosan/Genipin/Pluronic F127	Nanostrucutred	Rabbits	Quercetin	[56], 2020
	PLGA-PEG-PLGA	Thermo-responsiveness	Optic nerve crushed rats	Ciliary neurotrophic factor/triamcinolone acetonide	[45], 2024
Bone and cartilage	Hyaluronic-acid	Double network/self-healing	Periodontitis rats	Rg1 and amelogenin	[46], 2021
	Quaternized Chitosan/Pluronic F127	Self-healing	Rotator cuff rabbits	Magnesium ions	[48], 2020
	Chitosan/reduced graphene oxide	NIR light responsiveness	Calvarial defect osteoporotic rats	Teriparatide	[57], 2021
	Silk fibroin/sodium alginate	pH- responsiveness	Chronic osteomyelitis rats	Teicoplanin/phenamil	[58], 2022
	Hyaluronic-acid	Self-healing	Femoral fracture mice	Exosomes/APY29	[59], 2022
	Sulfated hyaluronic acid/β-cyclodextrin	Self-healing	Articular cartilage defect rabbits	TGF-β1 Kartogenin	[60], 2023
	Hyaluronan/pNIPAM	Thermo-responsiveness	Osteoporotic rats	Zoledronic acid/bone morphogenetic protein 2	[47], 2024
	Chitosan/waterborne polyurethane cryogel	Self-healing	Osteochondral defect rabbits	Rho-kinase inhibitor Y27632 /dexamethasone	[49], 2024
Cardiovascular	Glycidyl methacrylated hyaluronic acid	Mechanical sensitivity	Rabbits	Simvastatin	[50], 2020
	Silicate nanodisks/polyethylene glycol	Self-healing	Intrapericardial injury rabbit	Dexamethasone	[61], 2021
	GelPB/polyvinyl alcohol	pH- and ROS-responsiveness	Coronary artery ligation rats	Natriuretic peptide/semaphorin3A	[51], 2025
	Dialdehyde hyaluronic acid/carbohydrazide-modified collagen/MWCNT	pH-responsiveness	Myocardial ischemia–reperfusion injury rats	Metformin/exosomes	[52], 2025
Intestinal	5’-GMP/NAP	pH-responsiveness	Mice with tumor	Doxorubicin	[62], 2023
Lung	Pectin carboxymethyl cellulose	Self-healing	Mice with lung cancer	Silibinin	[63], 2023
Renal	Hyaluronic-acid/Pluronic F127	Hierarchical/Self-healing	Renal fibrosis mice	Celastrol/anti-TGFβ	[64], 2021
Uterus	Polydopamine/agarose	NIR light responsive	Rats with endometriosis	Letrozole	[65], 2023
Colon	Carboxymethyl chitosan/ chondroitin sulfate/ Pluronic F127	Thermo-responsiveness	Ulcerative colitis mice	Periplaneta americana extracts	[66], 2023
Tumor	Carboxymethyl chitosan/Pluronic F127	pH/thermal responsiveness	EMT6 tumor-bearing mice	Indocyanine green/doxorubicin	[67], 2023
	Phenylboronic acid/ polyvinyl alcohol/TiO_2_ nanosheet	pH/ROS/ultrasound responsiveness	Mice with triple-negative breast cancer tumor	Anti PD-L1/Ca^2+^	[68], 2023
	Hyaluronic acid/PF 127/Ti-MOF-Au	Sonosensitive/ROS- responsiveness	Bagg Albino tumor mice	Polyethylene glycol–thioketal–doxorubicin	[53], 2024

poly(lactic-co-glycolic acid) as PLGA, polyethylene glycol as PEG, poly(N-isopropylacrylamide) as PNIPAM, poly (ethylene glycol) diacrylate as PEG-DA, 5’-guanosine monophosphate as 5’-GMP, 1,4,5,8-naphthalene tetracarboxylic acid tetra potassium salt as NAP, and reactive oxygen species as ROS, fluorophenylboronic acid-modified gelatin as GelPB, polyvinyl alcohol as PVA, multi-walled carbon nanotubes as MWCNT

### Central nervous system

Blood–brain barriers are natural barriers that protect the brain, control CNS homeostasis and immune environments, and regulate molecular exchange between the CNS and blood. However, the same biological mechanisms that protect the brain also limit transvascular administration. Accordingly, hydrogel delivery therapies have been used for treating three major categories of brain diseases, including brain tumors, CNS injuries, and neurodegenerative diseases (such as Parkinson’s, Alzheimer's diseases, and so on). For example, a self-healing hydrogel composed of phenolic chitosan and a micellar crosslinker with a modulus comparable to that of the brain was developed [41]. The hydrogel showed asynchronous dual-drug encapsulation that can be potentially applied to treat the intracerebral hemorrhagic stroke. Two clinical drugs were released in different kinetics to meet the needs of different pathological stages of stroke. In a rat study, behavioral improvement and histological neuroangiogenesis were clearly observed [34]. Another self-healing hydrogel based on carboxymethyl chitosan and aldehyde-functionalized Pluronic F127 containing black phosphorus-methylene blue was developed to prevent the accumulation of hyperphosphorylated tau protein for treating Alzheimer's disease by intranasal delivery. The hydrogel suppressed tau neuropathology, restored mitochondrial function, and alleviated neuroinflammation, thus inducing cognitive improvements in Alzheimer's disease mouse models [42]. The usage of soft (~ 0.1–1 kPa) and injectable hydrogels is necessary to carry drugs deep into brain tissue lesions. Also, high concentrations of reactive oxygen species (ROS) and inflammatory microenvironments are often associated with neurodegeneration or damage, making treatment more complicated. ROS/enzyme-responsive hydrogel release mechanisms can promote local release of brain drugs. The stimuli-responsive hydrogel in these studies is often designed to have similar properties to the extracellular matrix of the brain, taking a transformative approach in neurotherapeutics that offers stage-specific drug delivery while minimizing systemic side effects.

### Ophthalmology

In ocular drug administration, there are numerous ocular barriers in both the anterior chamber and the posterior segment of the eye. Currently, five commonly used routes for ocular drug delivery include topical, subconjunctival, intravitreal (IVT), subretinal, and suprachoroidal injections. IVT injection is the most frequently employed method for posterior segments targeting clinical practice. However, excessively frequent IVT injections not only impede patient compliance with treatment but also significantly increase the risk of adverse reactions and side effects such as retinal detachment, intraocular infection, cataract formation, hemorrhage, and increased intraocular pressure [43]. The IVT release of therapeutics via hydrogel satisfies the need for effective and less frequent dosing. A thermos-responsive hydrogel composed of poly(lactic-co-glycolic acid) microspheres was used as a novel DDS to deliver aflibercept. This smart hydrogel underwent rapid transformation from a liquid state upon intravitreal injection to a viscous elastic solid at the physiological temperature. Evaluation in an animal model of adult macaques (Macaca mulatta) demonstrated the controlled and sustained release of aflibercept for up to 6 months without any adverse reactions [44]. In another work, an injectable thermosensitive hydrogel drug delivery system that mimics the mechanical properties of the vitreous body and enables sustained release of ciliary neurotrophic factor and triamcinolone acetonide was developed to promote the retinal ganglion cell survival and axon regeneration in a traumatic optic neuropathy model [45]. The eye has multiple barriers, resulting in extremely short retention time (< 30 min) of drug drops or injections and low bioavailability. Both the retina and cornea are highly sensitive tissues with low tolerance to foreign body stimulation. Hydrogels need to have high transparency, optical stability, high adhesion, and wet surface stability (bioinertness). Temperature- or ion-responsive hydrogels with transparency and adhesion can achieve sustained release by adjusting the original surface charge while reducing visual interference.

### Orthopedics and dentistry

Even in hard tissues such as bones and teeth, highly flexible drug-loaded smart hydrogels still have a role to play. A hyaluronan-based hydrogel formed by a dynamic crosslinked network of dynamic Schiff base and dynamic coordination bonds encapsulated and releasing ginsenoside Rg1 and amelogenin within 7 d successfully promoted periodontal regeneration in the treatment of periodontitis in a rat model in vivo [46]. In another study, a thermo-responsive hyaluronan/poly(N-isopropylacrylamide) hydrogel was employed as a local delivery system to enhance the implant stability in an osteoporotic rat model. The combined delivery of zoledronic acid and bone morphogenetic protein 2 induced both an osteogenic response and prevented bone resorption [47]. Moreover, as a soft material, hydrogel is often used as an interface filler between the tissue and bone to play a buffering role. A multifunctional self-healing quaternized chitosan/micelles hydrogel achieved in situ and customized release of Mg^2+^ ions. This hydrogel showed good injectability and adhesive properties, enabled easier and stable delivery at irregularly shaped tendon-bone interfaces, and successfully regenerated the fibrocartilaginous interface in a vivo rabbit rotator cuff tear model in vivo [48]. Recently, a type of “cryogel material” derived from self-healing hydrogel has been developed to as a cartilage matrix with controllable porosity and high modulus. The cryogel was prepared based on chitosan-polyurethane self-healing hydrogel using a freezing–thawing process to obtain a multilayer cryogel, delivering Rho-associated protein kinase inhibitor Y27632 and dexamethasone to regenerate the osteochondral defects in rabbits [49]. To further advance the application of smart hydrogels in orthopedics and dentistry, specific mechanical and biological conditions should be considered. For hard bone tissues, the presence of a mineralized barrier, high stiffness, and the requirement to endure high compressive loads necessitate robust mechanical performance of the hydrogel system. For cartilage repair, smart hydrogels should withstand sustained compressive and shear forces (> 1 MPa) within the joint cavity and facilitate stem cell paracrine signaling and lineage-specific differentiation. These biomechanical parameters, especially when applied to the interface between different tissues, are critical for designing hydrogels tailored to the complex needs of orthopedic and dental regeneration.

### Cardiovascular

Atherosclerosis, myocardial infarction (MI), heart failure, and other cardiovascular diseases are notorious for high disability and mortality rates globally. The development of hydrogels provides a new approach to address this issue and demonstrates the potential for treating cardiovascular diseases. The unique porous structure of hydrogels is particularly important for cardiac tissue engineering due to the high transport of nutrients and metabolism of paracrine factors. A hydrogel based on high molecular weight hyaluronic acid modified with glycidyl methacrylate exhibits mechanical sensitivity for drug release under long-term circulation conditions in narrow blood vessels and can target inflammatory macrophages at thrombus sites. The hydrogel loaded with simvastatin revealed effective simvastatin release in a rabbit model and precise targeting in and apoE-/- mice model, marking the first hydrogel successfully applied in atherosclerosis research [50]. Biocompatible, adhesive, and highly conductive hydrogel cardiac patches are expected to find clinical therapeutic applications in myocardial infarction (MI). A smart pH- and ROS-responsive fluorophenylboronic acid-modified gelatin/polyvinyl alcohol hydrogel loaded with c-type natriuretic peptide (CNP) and Sema3A was developed for post-infarction repair, offering a promising therapeutic strategy for addressing both malignant arrhythmia and heart failure post-MI [51]. Furthermore, a pH-responsive conductive hydrogel was designed for the intelligent release of metformin and exosomes to enhance cardiac repair of MI. The study confirmed that the hydrogel treatment reduced the infarct size, cardiac fibrosis, and incidence of arrhythmia, while improving ventricular ejection fraction and facilitating the restoration of cardiac function after MI [52].

For the effective application of hydrogels in cardiology, several physiological and pathological conditions must be addressed. The myocardium is subjected to high pulsatility and cyclic strain, which requires hydrogels to possess dynamic mechanical compliance and elasticity. In addition, the ischemic and post-infarction microenvironment is characterized by elevated levels of matrix metalloproteinases and ROS, thus requiring hydrogels to have enzymatic or redox degradation properties for controlled release and biodegradation. For post-myocardial infarction treatments, patch-based hydrogel systems are often preferred to allow for local delivery and mechanical support of the infarcted area. Furthermore, in the case of atherosclerosis or thrombosis, hydrogels must be able to target and penetrate ischemic thrombi, crossing complex vascular geometries and pathological barriers. Customization of hydrogels to address these biomechanical and biochemical challenges is essential for the next generation of cardiovascular regenerative therapies.

### Clinical trials and productization

Smart hydrogels show great potential in pre-clinical research. However, there are still many challenges in the actual advancement to the clinical and market stages, including four aspects: (1) the biocompatibility and stability of materials; (2) the controllability and reproducibility of drug release behavior; (3) the feasibility of clinical operations; and (4) the difficulty of scaling up and standardizing the manufacturing process.

At present, a few drug-containing smart hydrogel products have been successfully launched on the market, one of which is Jelmyto^®^. It is a thermo-responsive hydrogel that is liquid at room temperature. After being injected into the upper urinary tract, it turns into a gel state at human body temperature and continuously releases the chemotherapy drug (mitomycin-C) for the treatment of low-grade upper urinary tract urothelial carcinoma. Its intelligence is reflected in the temperature-triggered phase transition and sustained release ability, without the need for multiple administrations or surgical removal. It was approved by the FDA in 2020. Another example is ACUVUE^®^ Theravision, which is the first product that combines an antiallergic drug (ketotifen) with contact lenses. It has a humidity-responsive drug release design and presents mechanism to slowly release the drug when the lens contacts the moist environment on the surface of the eye, effectively relieving the symptoms of allergic conjunctivitis. It is currently available in Japan, Canada, and other places.

In the meantime, some smart hydrogels have failed in commercialization. OncoGel® uses thermo-sensitive hydrogel as a carrier to locally deliver the anticancer drug (paclitaxel), which has been used to treat esophageal cancer and brain tumors. Although it demonstrated promise in Phase I/II clinical trials, the program was ultimately discontinued during Phase III due to limited therapeutic efficacy, challenges in managing local drug toxicity, procedural complexity, and lack of industrial momentum, highlighting the significant hurdles in commercializing such smart drug-loaded hydrogels.

### Expectations and challenges

The drug in situ delivery through smart hydrogels has attracted widespread attention, especially in regenerative medicine engineering and the local release of refractory diseases in deep tissue sites. Smart hydrogels can respond to certain physiological stimuli to release drugs in expected scenarios, but there are still challenges in different aspects. The introduction of some intelligent properties depends on external factors rather than the hydrogel itself, such as the introduction of MWCNT for electrical responsiveness and Ti-MOF-Au for sound responsiveness. The introduction of these additives may require the re-evaluation of the compatibility of the whole system and the safety of the degradation products. Secondly, biodegradable hydrogels exhibit time-dependent properties, including stability, swelling, and viscoelasticity, leading to attenuation of performance in different stages during prolonged in situ applications. This highlights the critical importance of defining appropriate initial and boundary conditions during the design of smart hydrogel systems. At the same time, due to individual differences in patients, the choice between large-scale manufacturing and customized production is particularly important and directly affects the production cost. The final point to note is the appropriate exit mechanism (degradation or surgical removal) for degradable smart hydrogels. It is necessary to design a reasonable degradation time and detect the toxicity of the degradation products. Meanwhile, the non-metabolizable smart hydrogel components require rigorous evaluation of dosage and exit mechanisms. Research efforts are mainly directed toward optimizing the compatibility between drugs, hydrogel carriers, and host tissues for improved therapeutic outcomes. In summary, the successful clinical translation of smart hydrogel products may depend on focusing efforts toward single-stimulus designs, low-toxicity and biodegradable materials, streamlined clinical procedures, and precise targeting of specific disease conditions. Such a strategy can reduce the difficulty of supervision and operation, can gain greater acceptance from medical institutions and patients, and can pave the way for smart hydrogels to transition from the laboratory to clinical and industrial applications.

## Conclusion

With the continuous improvement and development of various smart hydrogel systems, an increasing number of delivery strategies are being designed for applications in various organs and tissues. Smart hydrogels, as an excellent extracellular matrix candidate for temporary filling and tissue repair, can effectively integrate cells, bioactive factors, and physical factors. Smart hydrogels demonstrate advantages for in vivo and in situ drug delivery. They also provide precise spatial control to reduce side effects and tunable properties such as biodegradation, bioadhesiveness, injectability, and tailored mechanical strength for various tissues. Compared to oral or intravenous administration, systemic drug delivery often suffers from dilution in the bloodstream, metabolic clearance, and nonspecific distribution, leading to low therapeutic efficiency and high systemic toxicity. Smart hydrogels, on the other hand, can be triggered by stimuli such as temperature, pH, or enzymes to form a stable drug reservoir at the target site, enabling sustained and controlled release. This approach significantly enhances local therapeutic efficacy while minimizing off-target effects, making it especially suitable for diseases requiring long-term or high-concentration local treatment, such as tumors and chronic inflammation. At the same time, smart hydrogels offer promising solutions to the tissue-specific challenges of drug delivery. Different tissues present unique physiological barriers and microenvironments. For example, intracranial injection of smart hydrogels can establish localized drug release platforms that effectively bypass the blood–brain barrier. In the eye, immune privilege and tear clearance limit drug retention time, but hydrogel systems adhering to the cornea or vitreous cavity can achieve prolonged and stable delivery. However, there are still many challenges that need to be evaluated and addressed, including standardization of processes, customized production conditions, interaction between drug molecules and hydrogel matrices, drug encapsulation dosage windows, and usage and metabolism cycles. In the meantime, emerging technologies such as 4D bioprinting, ultrasound- or mechanically triggered systems, and multi-responsive nanocomposite hydrogels are enabling dynamic tissue reconstruction and precise spatiotemporal control of drug release, further expanding the capabilities of smart hydrogel platforms. Looking forward, there is an expectation for more exploration into the new generation smart hydrogels, as well as more animal models and clinical experiments to evaluate strategies involving drug-loaded smart hydrogels.

## Data Availability

No datasets were generated or analysed during the current study.
